# Associations of complete blood cell count-derived inflammatory biomarkers with asthma and mortality in adults: a population-based study

**DOI:** 10.3389/fimmu.2023.1205687

**Published:** 2023-07-28

**Authors:** Junhua Ke, Fushan Qiu, Wenxi Fan, Songqing Wei

**Affiliations:** ^1^ Department of Geriatric Rehabilitation, Rehabilitation Hospital affiliated to Fujian University of Traditional Chinese Medicine, Fuzhou, Fujian, China; ^2^ Fujian Key Laboratory of Rehabilitation Technology, Fuzhou, Fujian, China

**Keywords:** CBC-derived inflammatory biomarkers, asthma, mortality, RSF, NHANES

## Abstract

**Objective:**

This study aims to assess the associations of complete blood cell count (CBC)-derived inflammatory biomarkers with the prevalence of asthma and mortality.

**Methods:**

Data was collected from the 1999-2018 National Health and Nutrition Examination Survey (NHANES). Mortality was identified using the National Death Index until December 31, 2019. The study analyzed the relationship between CBC-derived inflammatory biomarkers, including neutrophil-to-lymphocyte ratio (NLR), platelet-to-lymphocyte ratio (PLR), monocyte-to-lymphocyte ratio (MLR), systemic inflammatory response index (SIRI), and systemic immune-inflammation index (SII), and the prevalence of asthma using multiple logistic regressions. To assess the significance of CBC-derived inflammatory biomarkers in predicting all-cause and respiratory disease mortality in asthma patients, Cox proportional regressions and the random survival forest (RSF) analysis were utilized.

**Results:**

A total of 48,305 participants were included, with a mean age of 47.27 ± 0.18 years and 49.44% male. Among them, 6,403 participants had asthma, with a prevalence of 13.28%. The all-cause and respiratory disease deaths at a median follow-up of 8.2 (4.5, 12.8) years were 929 and 137 respectively. After adjusting for confounders, the prevalence of asthma was found to be positively associated with NLR, PLR, MLR, SIRI and SII. Compared to the lowest quartile, the highest quartile of NLR (HR=1.765 [1.378-2.262]), MLR (HR=1.717 [1.316-2.241]), SIRI (HR=1.796 [1.353-2.383]) and SII (HR=1.432 [1.141-1.797]) were associated with an increased risk of all-cause mortality. These associations were more pronounced in respiratory disease mortality of asthma patients. RSF analysis showed that MLR had the highest predictive value for all-cause and respiratory disease mortality in adults with asthma. The sensitivity analysis demonstrated the stability of our results.

**Conclusion:**

The findings suggest that CBC-derived inflammatory biomarkers are associated with a higher risk of all-cause and respiratory disease mortality in adults with asthma.

## 1 Background

//Asthma has become a severe public health problem, affecting people from childhood to old age (1). Approximately 300 million people in the world have asthma, with at least 250,000 deaths attributed to the disease each year (2). Inflammation plays a pivotal role in the process of developing asthma (3). Both local and systemic inflammation are involved in the development of asthma (4, 5). Neutrophils, in the early stages of asthma exacerbations, play a crucial role by releasing various pro-inflammatory mediators, thereby contributing to airway inflammation (6). Monocytes differentiate into macrophages, which are central to the chronic inflammation observed in asthma (7). Macrophages release inflammatory molecules like cytokines and chemokines, which attract and activate other immune cells, perpetuating the inflammatory response. Lymphocytes, particularly T-helper 2 (Th2) lymphocytes, also play a significant role in asthma (8). Th2 lymphocytes release cytokines that promote airway inflammation, mucus production, and bronchoconstriction (8). Consequently, inflammatory indicators appear to be effective biomarkers for assessing severity and potential therapeutic targets in patients with asthma (9). Current studies have reported that a variety of inflammation-related indicators might be related to outcomes in asthma patients (10, 11), but the optimal biomarker for asthma is unclear.

Recently, CBC-derived inflammatory biomarkers such as the neutrophil-to-lymphocyte ratio (NLR), platelet-to-lymphocyte ratio (PLR), monocyte-to-lymphocyte ratio (MLR), systemic inflammatory response index (SIRI), and systemic immune-inflammation index (SII) were used as prognostic factors in various diseases (12, 13). These biomarkers are based on two or three parameters related to neutrophils, lymphocytes, platelets, and monocytes. The NLR, SII, and SIRI have a predictive power of severe COVID-19 and invasive mechanical ventilation (IMV) support during hospitalization in Mexican COVID-19 patients (14). In addition, the WBC count, neutrophil count, dNLR, and SII were significantly associated with survival in COVID-19 patients (15). Similarly, in individuals diagnosed with community-acquired pneumonia, neutrophil-lymphocyte ratio has been associated with increased mortality risk (16). Furthermore, in chronic obstructive pulmonary disease (COPD) patients, elevated neutrophil-to-lymphocyte ratio (NLR), platelet-to-lymphocyte ratio (PLR) values have been linked to higher mortality rates (17, 18). Additionally, the SII, another CBC-derived biomarker, has demonstrated its potential in predicting mortality among middle-aged and elderly individuals with COPD and asthma (19).

Asthma is a chronic inflammatory disorder primarily affecting the airways. The underlying immune and inflammatory mechanisms in asthma may differ from those in other diseases, such as infectious diseases or systemic inflammatory conditions. Thus, the inflammatory profiles and immune responses can vary among different subgroups of asthma patients. Furthermore, the relationship between CBC-derived inflammatory biomarkers and survival has not been comprehensively evaluated in patients with asthma. Therefore, using data from the 1999-2018 National Health and Nutrition Examination Survey (NHANES), this study examined the association between CBC-derived inflammatory biomarkers and the prevalence of asthma, as well as the mortality in participants with asthma. Our study aimed to provide a valuable prognostic indicator and guide the individual treatment of asthma.

## 2 Materials and methods

### 2.1 Study population

Data were obtained from the NHANES, which is a national survey that collected nutritional and health status of children and adults (20). This project is conducted by the National Center for Health Statistics (NCHS) at the Centers for Disease Control and Prevention (CDC) and focuses on collecting basic physical and biochemical examinations and other medically relevant information. The National Center for Health Statistics (NCHS) Research Ethics Review Board approved the research protocols, and all participants gave informed consent.

In this study, we analyzed data from the 1999-2018 NHANES. We excluded participants under the age of 20 and those with missing asthma assessment data. Participants who were pregnant, lacking data on CBC parameters, and had no follow-up information were also excluded.

### 2.2 Assessment of CBC-derived inflammatory biomarkers

The complete blood count (CBC) is measured using automated hematology analyzers, which count the number of different blood cells (red blood cells, white blood cells, and platelets) in given volume of blood. We calculated the SIRI, SII, NLR, PLR, and MLR as follows: SIRI = neutrophil counts × monocyte counts/lymphocyte counts, SII = platelet counts × neutrophil counts/lymphocyte counts, NLR = neutrophil counts/lymphocyte counts, PLR = platelet counts/lymphocyte counts, MLR = monocyte counts/lymphocyte counts (12, 13).

### 2.3 Assessment of asthma

By using a self-administered questionnaire, NHANES collected information on asthma and associated symptoms (21). Patients who answered affirmatively to the following two questions were defined as having current asthma: “Has a doctor or other health professional ever told you that you have asthma?” and “Do you still have asthma?”. Patients who gave negative answers to these two questions were used as the control group.

### 2.4 Assessment of mortality

Study participants who had died were identified by linkage to the National Death Index (NDI). As of December 31st, 2019, we obtained all-cause and respiratory disease mortality records for participants via the 2019 Linked Mortality File (LMF), which reports the latest associations made between selected NCHS surveys and the NDI.

### 2.5 Covariates

Information regarding participants’ baseline data was collected through questionnaires and laboratory tests, including age (years), sex (male or female), race/ethnicity (Mexican American, other Hispanic, non-Hispanic white, non-Hispanic black, or other race), educational attainment (below high school, high school, or above high school), and body mass index (<25.0, 25.0-29.9, or >29.9 kg/m^2^). Income was measured using the poverty-income ratio (PIR; the ratio of family income divided by a poverty threshold specific for family size using guidelines from the US Department of Health and Human Services) and categorized as ≤1.0, 1.1-3.0, and >3.0 (22). Never smokers were identified as those who reported smoking <100 cigarettes over the course of their lifetime. Those who smoked >100 cigarettes in their lifetime were classified as current smokers, and those who smoked >100 cigarettes and had quit smoking were labeled as former smokers (23). Drinking status was classified as nondrinker, low-to-moderate drinker (<2 drinks/day in men and <1 drink/day in women), or heavy drinker (≥2 drinks/day in men and ≥1 drinks/day in women) (23). Physical activity was divided into three groups: inactive (no leisure-time physical activity), insufficiently active (moderate activity 1–5 times per week with metabolic equivalent of task [MET] 3–6 or vigorous activity 1–3 times per week with MET >6), and active (those who had more moderate or vigorous activity than the previously mentioned) (23–25). The energy intake (kcal/day) was calculated by averaging two values from two 24-hour recall interviews. Information on the prevalence of hypertension and diabetes was retrieved using self-reported questionnaires.

### 2.6 Statistical analysis

Normally distributed continuous variables were described as means ± standard errors (SEs), and continuous variables without a normal distribution were presented as medians (interquartile range [IQR]). Categorical variables were presented as numbers (percentages). Continuous variables are compared using Student’s t-test (normal distribution) or the Mann-Whitney U test (non-normal distribution). Categorical variables are compared using the chi-square test. Missing values for covariates were filled using the imputation approach, which was based on a “mice” package of the Random Forest algorithm. We performed all statistical analyses using R software (version 4.2.0).

A multiple logistic regression model was employed to ascertain the adjusted odds ratios (ORs) and 95% confidence intervals (CIs) of the association between CBC-derived inflammatory biomarkers and the prevalence of asthma. A multiple COX regression was implemented to calculate adjusted hazard ratios (HRs) and 95% CIs in relation to all-cause and respiratory disease mortality of participants with asthma. To explore the dose–response curves between CBC-derived inflammatory biomarkers and mortality in asthma patients, restricted cubic spline regression analysis was performed. The knots were placed at each exposure variable’s 10th, 50th and 90th percentiles.

Spearman’s correlation analysis was used to calculate the correlation coefficients among CBC-derived inflammatory biomarkers and CBC. A random survival forest method was used to compare the value of CBC-derived inflammatory biomarkers in predicting all-cause and respiratory disease mortality in asthma patients. Stratified analysis was conducted to assess the associations between quartiles of CBC-derived inflammatory biomarkers levels and all-cause and respiratory disease mortality by smoking among adults with asthma in NHANES 1999–2018.

Multiple sensitivity analyses were performed to evaluate the robustness of the results. Firstly, we further adjusted for anti-asthmatic medications such as bronchodilators, inhaled corticosteroids, or other anti-asthmatic drugs, as well as lung function parameters including forced vital capacity (FVC) and forced expiratory volume 1st second (FEV1). Secondly, to minimize the potential for reverse causation bias, participants who died within the first two years of follow-up were excluded from the study. Additionally, participants with a history of cancer, HIV infection, or respiratory infections at baseline were also excluded from the study. Cox regression analysis was then performed to assess the relationship between CBC-derived inflammatory biomarkers and mortality in asthma participants. Lastly, participants with self-reported chronic obstructive pulmonary disease (COPD) at baseline were excluded from the study, and the relationship was analyzed once again.

## 3 Results

### 3.1 Characteristics of study participants

Between 1999 and 2018, 48,305 adults participated in the NHANES. We removed participants younger than 20 and missing data on asthma assessment (n=46,235). Subsequently, we excluded participants who were pregnant (n=1,317), and those who lacked data on CBC parameters (n=5,459). In addition, the eleven participants with asthma whom we failed to interview were excluded, resulting in a total of 6,403 participants with asthma for survival analysis (**Figure S1**).

**Table 1** displays the baseline characteristics of adults with CBC-derived inflammatory biomarkers in the 1999–2018 NHANES. The study population had a mean age of 47.27 ± 0.18 years, was 49.44% male, and was mainly non-Hispanic white (44.57%). The medians of NLR, PLR, MLR, SIRI and SII were 2.00 [IQR 1.51, 2.62], 121.43 [IQR 96.45, 153.13], 0.26 [IQR 0.21, 0.33], 1.06 [IQR 0.73, 1.53] and 487.18 [IQR 353.69, 680.77], respectively. There were 6,414 (13.28%) participants have asthma. Compared to non-asthmatic participants, they were more likely to be younger non-Hispanic White females, to have higher education and lower income levels, to be current smokers, low-to-moderate drinkers, physically inactive, to have a higher BMI, and to have higher prevalence of hypertension (*P*<0.05). The counts of white blood cell (WBC), neutrophils, monocyte, lymphocyte, and platelet were significantly higher in asthma patients (*P*<0.05). Among all CBC-derived indicators, only SIRI and SII showed significant differences between participants with and without asthma.

**Table 1:** Baseline characteristics of adults with CBC-derived inflammatory biomarkers in NHANES 1999–2018.

Characteristics	Total (n=48305)	Asthma		*P* value
		No (n=41891)	Yes (n=6414)	
Age, years	47.27(0.18)	47.58(0.19)	45.36(0.29)	<0.001
Male, %	23883(49.44)	21171(49.88)	2712(41.33)	<0.001
Race/ethnicity, %				<0.001
Mexican American	8429(17.45)	7773(8.59)	656(4.78)	
Other Hispanic	4011(8.3)	3420(5.59)	591(5.97)	
Non-Hispanic White	21530(44.57)	18444(68.44)	3086(70.45)	
Non-Hispanic Black	9883(20.46)	8356(10.49)	1527(12.19)	
Other race	4452(9.22)	3898(6.89)	554(6.61)	
Education level, %				0.001
Below high school	13160(27.24)	11654(17.64)	1506(15.79)	
High school	11173(23.13)	9732(24.28)	1441(22.79)	
Above high school	23972(49.63)	20505(58.08)	3467(61.42)	
Family PIR, %				<0.001
≤1.0	10060(20.83)	8456(13.81)	1604(18.18)	
1.1–3.0	20472(42.38)	17865(36.52)	2607(35.91)	
>3.0	17773(36.79)	15570(49.67)	2203(45.91)	
Smoking status, %				<0.001
Never smoker	26056(53.94)	22919(54.34)	3137(49.45)	
Former smoker	12059(24.96)	10363(24.52)	1696(26.05)	
Current smoker	10190(21.1)	8609(21.14)	1581(24.51)	
Drinking status, %				0.020
Nondrinker	11192(23.17)	9810(18.93)	1382(17.65)	
Low-to-moderate drinker	33185(68.7)	28658(71.35)	4527(73.57)	
Heavy drinker	3928(8.13)	3423(9.71)	505(8.78)	
Body mass index, %				<0.001
<25.0 kg/m^2^	14142(29.28)	12512(31.23)	1630(28.34)	
25.0-29.9 kg/m^2^	16307(33.76)	14453(34.02)	1854(28.89)	
>29.9 kg/m^2^	17856(36.97)	14926(34.75)	2930(42.77)	
Physical activity, %				0.020
Inactive	13456(27.86)	11631(22.18)	1825(23.97)	
Insufficiently active	17742(36.73)	15482(40.18)	2260(38.12)	
Active	17107(35.41)	14778(37.64)	2329(37.91)	
Total energy intakes, kcal/day	2015.00(1491.00,2698.00)	2020.00(1496.00,2692.00)	1994.00(1467.00,2738.00)	0.490
Self-reported hypertension, %	17098(35.4)	14445(30.02)	2653(35.03)	<0.001
Self-reported diabetes, %	6017(12.46)	5040(8.71)	977(10.68)	0.480
CBC count, 10^3^/μL				
White blood cell	6.90(5.70,8.40)	6.90(5.70,8.40)	7.20(5.90,8.70)	<0.001
Neutrophils	4.00(3.20,5.20)	4.00(3.10,5.10)	4.20(3.20,5.40)	<0.001
Monocyte	0.50(0.40,0.70)	0.50(0.40,0.70)	0.50(0.40,0.70)	0.010
Lymphocyte	2.00(1.60,2.50)	2.00(1.60,2.50)	2.10(1.70,2.50)	0.002
Platelet	246.00(210.00,291.00)	245.00(209.00,290.00)	251.00(213.00,297.00)	<0.001
CBC-derived indicators				
NLR	2.00(1.51,2.62)	2.00(1.50,2.61)	2.00(1.54,2.65)	0.050
PLR	121.43(96.45,153.13)	121.50(96.39,152.94)	121.18(96.82,154.38)	0.600
MLR	0.26(0.21,0.33)	0.26(0.21,0.33)	0.26(0.21,0.33)	0.420
SIRI, 10^3^/μL	1.06(0.73,1.53)	1.05(0.73,1.52)	1.08(0.75,1.58)	0.010
SII, 10^3^/μL	487.18(353.69,680.77)	483.85(351.91,676.67)	505.87(366.32,705.15)	<0.001

PIR, poverty income ratio; NLR, neutrophil-to-lymphocyte ratio; PLR, platelet-to- lymphocyte ratio; MLR, monocyte-to-lymphocyte ratio; SIRI, systemic inflammatory response index; SII, systemic immune-inflammation index; CBC, complete blood cell. Normally distributed continuous variables are described as means ± SEs, and continuous variables without a normal distribution are presented as medians [interquartile ranges]. Categorical variables are presented as numbers (percentages). N reflect the study sample while percentages reflect the survey-weighted.

Over a median follow-up of 8.2 (4.5, 12.8) years, there were 929 (4.5%) all-cause deaths among 6,403 adults with asthma **(** **Table S2** **)**. Compared to survivors, those who died of all causes were more likely to be older non-Hispanic White patients, to have lower education and income levels, to be current smokers, heavy drinkers, physically inactive, to have a higher BMI, and to have higher prevalence of hypertension and diabetes (*P*<0.05). In addition, all-cause deaths were more likely to have a higher value of all CBC-derived indicators (*P*<0.05).

### 3.2 Associations between CBC-derived indicators and the prevalence of asthma

All CBC-derived indicators were classified into four groups and assessed for their association with the prevalence of asthma (**Table 2**). The crude model revealed positive correlations between CBC-derived indicators (NLR, SIRI, and SII) and the prevalence of asthma. After adjusting for age, sex, and race, this relationship remained statistically significant.

**Table 2:** OR (95% CIs) of the prevalence of asthma according to quartiles of complete blood cell (CBC)-derived inflammatory biomarkers among adults in NHANES 1999–2018.

	Quartiles of CBC-derived inflammatory biomarkers levels				*P*_trend_
	Quartile 1	Quartile 2	Quartile 3	Quartile 4	
NLR					
Range	<1.43	1.43-1.91	1.92-2.56	>2.56	
Crude	1.00 [Reference]	1.036(0.939,1.144)	1.040(0.940,1.151)	1.113(1.004,1.235)	0.037
Model 1	1.00 [Reference]	1.068(0.966,1.180)	1.077(0.971,1.195)	1.202(1.079,1.339)	<0.001
Model 2	1.00 [Reference]	1.061(0.958,1.177)	1.042(0.937,1.158)	1.142(1.024,1.274)	0.02
PLR					
Range	<94.8	94.8-120.0	120.1-152.0	>152.0	
Crude	1.00 [Reference]	1.073(0.978,1.176)	0.969(0.874,1.076)	1.068(0.962,1.186)	0.458
Model 1	1.00 [Reference]	1.047(0.955,1.148)	0.943(0.848,1.048)	1.037(0.933,1.152)	0.808
Model 2	1.00 [Reference]	1.089(0.994,1.193)	1.000(0.899,1.112)	1.123(1.012,1.247)	0.088
MLR					
Range	<0.20	0.20-0.26	0.26-0.33	>0.33	
Crude	1.00 [Reference]	0.968(0.884,1.060)	0.919(0.830,1.017)	0.967(0.884,1.057)	0.495
Model 1	1.00 [Reference]	1.018(0.929,1.115)	1.000(0.903,1.107)	1.133(1.033,1.244)	0.007
Model 2	1.00 [Reference]	1.032(0.942,1.130)	1.019(0.922,1.125)	1.163(1.060,1.275)	0.001
SIRI					
Range	<0.68	0.68-1.02	1.03-1.50	>1.50	
Crude	1.00 [Reference]	1.064(0.973,1.163)	1.102(0.993,1.222)	1.147(1.043,1.262)	0.005
Model 1	1.00 [Reference]	1.115(1.017,1.223)	1.191(1.070,1.326)	1.289(1.165,1.426)	<0.0001
Model 2	1.00 [Reference]	1.080(0.985,1.184)	1.123(1.008,1.251)	1.169(1.056,1.293)	0.004
SII					
Range	<330.0	330.0-466.7	466.8-659.5	>659.5	
Crude	1.00 [Reference]	1.065(0.951,1.194)	1.162(1.041,1.297)	1.249(1.118,1.394)	<0.0001
Model 1	1.00 [Reference]	1.064(0.950,1.193)	1.159(1.038,1.295)	1.234(1.101,1.382)	<0.0001
Model 2	1.00 [Reference]	1.052(0.938,1.181)	1.119(0.999,1.252)	1.159(1.031,1.303)	0.008

Data are presented as OR (95% CI) unless indicated otherwise; Model 1 was adjusted as age (continuous), sex (male or female), and race/ethnicity (Mexican American, Other Hispanic, Non-Hispanic White, Non-Hispanic Black or Other); Model 2 was adjusted as model 1 plus education level (below high school, high school, or above high school), family poverty income ratio (≤1.0, 1.1–3.0, or >3.0), drinking status (nondrinker, low-to-moderate drinker, or heavy drinker), smoking status (never smoker, former smoker, or current smoker), BMI (<25.0, 25.0-29.9, or >29.9), physical activity (inactive, insufficiently active, or active), total energy intakes (in quartiles), self-reported diabetes (yes or no), and self-reported hypertension (yes or no).

In model 2, higher levels of NLR, PLR, MLR, SIRI, and SII were all significantly associated with an increased prevalence of asthma. Specifically, individuals in the fourth quartile of these inflammatory biomarkers had higher odds of having asthma than those in the first quartile. After adjusting for potential confounding factors, the odds ratios (ORs) and 95% confidence intervals (CIs) for the highest quartile compared to the lowest quartile were as follows: NLR (OR=1.142 [1.024-1.274], *P*_trend_=0.02), PLR (OR=1.123 [1.012-1.247], *P*_trend_=0.088), MLR (OR=1.163 [1.060-1.275], *P*_trend_=0.001), SIRI (OR=1.169 [1.056-1.293], *P*_trend_=0.004), and SII (OR=1.149 [1.031-1.303], *P*_trend_=0.008). We also analyzed the relationship between CBC parameters and the prevalence of asthma (**Table S1**). In the crude model, all CBC parameters were positively associated with the prevalence of asthma. After correcting for all confounding factors, we only found that WBC, neutrophils, monocyte were significantly associated with a higher prevalence of asthma.

### 3.3 Associations between CBC-derived indicators and mortality

**Figure 1:**
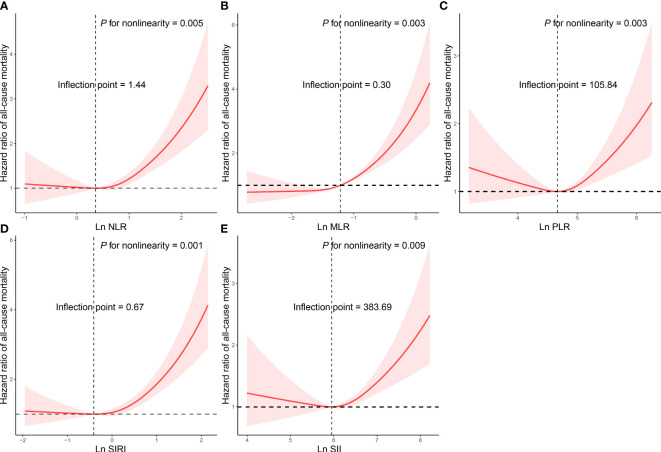
Restricted cubic spline analyses the association of complete blood cell count (CBC)-derived indicators (**A**: NLR; **B**: MLR; **C**: PLR; **D**: SIRI; **E**: SII) with all-cause mortality in adults with asthma. Adjusted for age (continuous), sex (male or female), race/ethnicity (Mexican American, Other Hispanic, Non-Hispanic White, Non-Hispanic Black or Other), education level (below high school, high school, or above high school), family poverty income ratio (≤1.0, 1.1–3.0, or >3.0), drinking status (nondrinker, low-to-moderate drinker, or heavy drinker), smoking status (never smoker, former smoker, or current smoker), BMI (<25.0, 25.0-29.9, or >29.9), physical activity (inactive, insufficiently active, or active), total energy intakes (in quartiles), self-reported diabetes (yes or no), and self-reported hypertension (yes or no).

As shown in **Table 3**, participants with asthma in quartile 4 had the highest risk of all-cause death. After multivariate adjustment in the Model 2, participants with asthma in quartile 4 of NLR (HR=1.765 [1.378-2.262]), MLR (HR=1.717 [1.316-2.241]), SIRI (HR=1.796 [1.353-2.383]) and SII (HR=1.432 [1.141-1.797]) remained significantly associated with an increased risk of all-cause mortality, compared with participants with asthma in quartile 4. CBC-derived indicators exhibited nonlinear associations with all-cause mortality of participants with asthma (*P* for nonlinearity<0.05, **Figure 1**). We also analyzed the relationship between CBC parameters and the risk of all-cause death in asthma patients (**Table S3**). After correcting for all confounding factors, we found that neutrophils (HR=1.364 [1.066-1.745]) and monocyte (HR=1.604 [1.208-2.129]) were significantly associated with a higher risk of all-cause mortality.

**Table 3:** HRs (95% CIs) of all-cause mortality according to quartiles of complete blood cell (CBC)-derived inflammatory biomarkers among adults with asthma in NHANES 1999–2018.

	Quartiles of CBC-derived inflammatory biomarkers levels				*P*_trend_
	Quartile 1	Quartile 2	Quartile 3	Quartile 4	
NLR					
Range	<1.43	1.43-1.91	1.92-2.56	>2.56	
No. deaths/total	73/3061	84/3066	119/3039	246/3078	
Crude	1.00 [Reference]	1.166(0.914,1.489)	1.169(0.908,1.504)	2.743(2.185,3.444)	<0.0001
Model 1	1.00 [Reference]	1.099(0.864,1.397)	1.141(0.886,1.470)	1.954(1.539,2.480)	<0.0001
Model 2	1.00 [Reference]	1.150(0.902,1.465)	1.083(0.847,1.384)	1.765(1.378,2.262)	<0.0001
PLR					
Range	<94.8	94.8-120.0	120.1-152.0	>152.0	
No. deaths/total	90/3065	112/3091	123/3032	197/3056	
Crude	1.00 [Reference]	0.795(0.606,1.041)	0.933(0.715,1.217)	1.275(1.003,1.619)	0.009
Model 1	1.00 [Reference]	0.756(0.599,0.954)	0.809(0.633,1.035)	0.965(0.774,1.205)	0.724
Model 2	1.00 [Reference]	0.854(0.679,1.075)	0.954(0.760,1.198)	1.168(0.934,1.460)	0.053
MLR					
Range	<0.20	0.20-0.26	0.26-0.33	>0.33	
No. deaths/total	61/3143	71/3008	93/2604	297/3489	
Crude	1.00 [Reference]	1.051(0.777,1.421)	1.332(0.996,1.782)	2.896(2.300,3.645)	<0.0001
Model 1	1.00 [Reference]	0.944(0.697,1.278)	1.009(0.745,1.366)	1.549(1.203,1.996)	<0.0001
Model 2	1.00 [Reference]	1.101(0.795,1.525)	1.141(0.837,1.555)	1.717(1.316,2.241)	<0.0001
SIRI					
Range	<0.68	0.68-1.02	1.03-1.50	>1.50	
No. deaths/total	62/3058	81/3066	126/3121	253/2999	
Crude	1.00 [Reference]	1.202(0.893,1.618)	1.639(1.259,2.135)	3.395(2.692,4.282)	<0.0001
Model 1	1.00 [Reference]	1.123(0.829,1.522)	1.436(1.064,1.937)	2.305(1.765,3.012)	<0.0001
Model 2	1.00 [Reference]	1.107(0.818,1.499)	1.313(0.980,1.760)	1.796(1.353,2.383)	<0.0001
SII					
Range	<330.0	330.0-466.7	466.8-659.5	>659.5	
No. deaths/total	87/3066	107/3056	121/3061	207/3061	
Crude	1.00 [Reference]	1.003(0.778,1.293)	1.158(0.915,1.465)	1.819(1.462,2.262)	<0.0001
Model 1	1.00 [Reference]	1.135(0.892,1.444)	1.257(0.980,1.612)	1.685(1.354,2.099)	<0.0001
Model 2	1.00 [Reference]	1.166(0.910,1.492)	1.158(0.902,1.487)	1.432(1.141,1.797)	0.003

Data are presented as HR (95% CI) unless indicated otherwise; Model 1 was adjusted as age (continuous), sex (male or female), and race/ethnicity (Mexican American, Other Hispanic, Non-Hispanic White, Non-Hispanic Black or Other); Model 2 was adjusted as model 1 plus education level (below high school, high school, or above high school), family poverty income ratio (≤1.0, 1.1–3.0, or >3.0), drinking status (nondrinker, low-to-moderate drinker, or heavy drinker), smoking status (never smoker, former smoker, or current smoker), BMI (<25.0, 25.0-29.9, or >29.9), physical activity (inactive, insufficiently active, or active), total energy intakes (in quartiles), self-reported diabetes (yes or no), and self-reported hypertension (yes or no).

**Figure 2:**
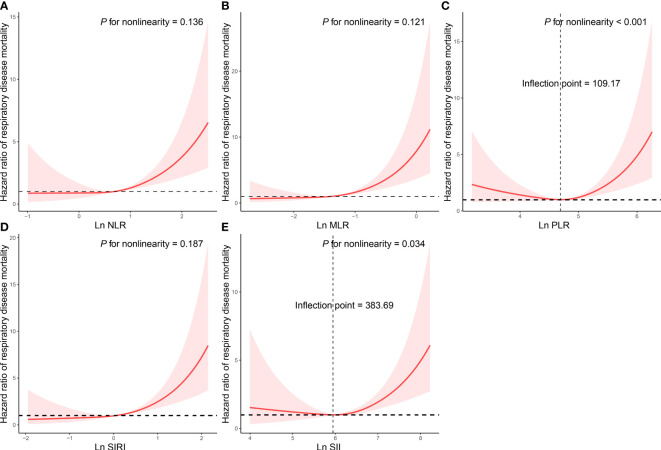
Restricted cubic spline analyses the association of complete blood cell count (CBC)-derived indicators (**A**: NLR; **B**: MLR; **C**: PLR; **D**: SIRI; **E**: SII) with respiratory disease mortality in adults with asthma. Adjusted for age (continuous), sex (male or female), race/ethnicity (Mexican American, Other Hispanic, Non-Hispanic White, Non-Hispanic Black or Other), education level (below high school, high school, or above high school), family poverty income ratio (≤1.0, 1.1–3.0, or >3.0), drinking status (nondrinker, low-to-moderate drinker, or heavy drinker), smoking status (never smoker, former smoker, or current smoker), BMI (<25.0, 25.0-29.9, or >29.9), physical activity (inactive, insufficiently active, or active), total energy intakes (in quartiles), self-reported diabetes (yes or no), and self-reported hypertension (yes or no).

Cox proportional regression results showed that NLR, MLR, SIRI and SII were associated with an increased risk of respiratory disease mortality in asthma patients (**Table 4**). After multivariate adjustment, the HR of NLR, MLR, SIRI and SII were 2.622, 2.812, 3.208, and 1.988, respectively (*P*<0.05). NLR, MLR, SIRI and SII exhibited linear associations with respiratory disease mortality of participants with asthma (*P* for nonlinearity>0.05, **Figure 2**). We also analyzed the relationship between CBC parameters and the risk of respiratory disease death in asthma patients (**Table S3**). After correcting for all confounding factors, we found that all CBC parameters were significantly associated with a higher risk of respiratory disease mortality (*P*<0.05).

**Table 4:** HRs (95% CIs) of respiratory disease mortality according to quartiles of complete blood cell (CBC)-derived inflammatory biomarkers among adults with asthma in NHANES 1999–2018.

	Quartiles of CBC-derived inflammatory biomarkers levels				*P*_trend_
	Quartile 1	Quartile 2	Quartile 3	Quartile 4	
NLR					
Range	<1.43	1.43-1.91	1.92-2.56	>2.56	
No. deaths/total	73/3061	84/3066	119/3039	246/3078	
Crude	1.00 [Reference]	0.828 (0.373-1.835)	1.411 (0.753-2.645)	4.610 (2.500-8.502)	<0.001
Model 1	1.00 [Reference]	0.774 (0.339-1.765)	1.365 (0.708-2.631)	3.121 (1.658-5.875)	<0.001
Model 2	1.00 [Reference]	0.756 (0.342-1.669)	1.096 (0.551-2.179)	2.622 (1.376-4.995)	<0.001
PLR					
Range	<94.8	94.8-120.0	120.1-152.0	>152.0	
No. deaths/total	90/3065	112/3091	123/3032	197/3056	
Crude	1.00 [Reference]	0.412 (0.200-0.847)	0.891 (0.436-1.820)	1.582 (0.853-2.932)	0.032
Model 1	1.00 [Reference]	0.385 (0.187-0.791)	0.742 (0.368-1.497)	1.133 (0.622-2.065)	0.203
Model 2	1.00 [Reference]	0.439 (0.223-0.864)	0.874 (0.460-1.660)	1.322 (0.781-2.239)	0.069
MLR					
Range	<0.20	0.20-0.26	0.26-0.33	>0.33	
No. deaths/total	61/3143	71/3008	93/2604	297/3489	
Crude	1.00 [Reference]	0.887 (0.400-1.966)	1.787 (0.846-3.774)	4.775 (2.557-8.918)	<0.001
Model 1	1.00 [Reference]	0.785 (0.348-1.770)	1.264 (0.590-2.708)	2.390 (1.236-4.622)	<0.001
Model 2	1.00 [Reference]	1.075 (0.465-2.486)	1.537 (0.746-3.165)	2.812 (1.507-5.246)	<0.001
SIRI					
Range	<0.68	0.68-1.02	1.03-1.50	>1.50	
No. deaths/total	62/3058	81/3066	126/3121	253/2999	
Crude	1.00 [Reference]	0.852 (0.375-1.936)	2.367 (1.133-4.945)	7.015 (3.550-13.863)	<0.001
Model 1	1.00 [Reference]	0.758 (0.331-1.736)	2.016 (0.919-4.419)	4.436 (2.187-8.999)	<0.001
Model 2	1.00 [Reference]	0.776 (0.333-1.809)	1.824 (0.839-3.965)	3.208 (1.578-6.522)	<0.001
SII					
Range	<330.0	330.0-466.7	466.8-659.5	>659.5	
No. deaths/total	87/3066	107/3056	121/3061	207/3061	
Crude	1.00 [Reference]	0.719 (0.343-1.503)	1.348 (0.657-2.768)	3.021 (1.670-5.466)	<0.001
Model 1	1.00 [Reference]	0.793 (0.373-1.682)	1.452 (0.687-3.068)	2.630 (1.415-4.889)	<0.001
Model 2	1.00 [Reference]	0.762 (0.344-1.689)	1.114 (0.536-2.315)	1.988 (1.043-3.789)	0.006

Data are presented as HR (95% CI) unless indicated otherwise; Model 1 was adjusted as age (continuous), sex (male or female), and race/ethnicity (Mexican American, Other Hispanic, Non-Hispanic White, Non-Hispanic Black or Other); Model 2 was adjusted as model 1 plus education level (below high school, high school, or above high school), family poverty income ratio (≤1.0, 1.1–3.0, or >3.0), drinking status (nondrinker, low-to-moderate drinker, or heavy drinker), smoking status (never smoker, former smoker, or current smoker), BMI (<25.0, 25.0-29.9, or >29.9), physical activity (inactive, insufficiently active, or active), total energy intakes (in quartiles), self-reported diabetes (yes or no), and self-reported hypertension (yes or no).

We performed a sensitivity analysis to investigate the robustness of our findings. Firstly, we excluded participants who died within two years of follow-up, and conducted a Cox regression analysis again. The results revealed that the relationship between CBC-derived indicators and all-cause and respiratory disease mortality persisted, as evidenced by the statistically significant association reported in **Table S4**. Furthermore, we conducted another sensitivity analysis by excluding participants who had a cancer history at baseline. The results of this analysis were also stable and consistent with our original findings, as demonstrated by the statistically significant association reported in **Table S5**.

### 3.4 Prognostic value of CBC-derived indicators

**Figure 3:**
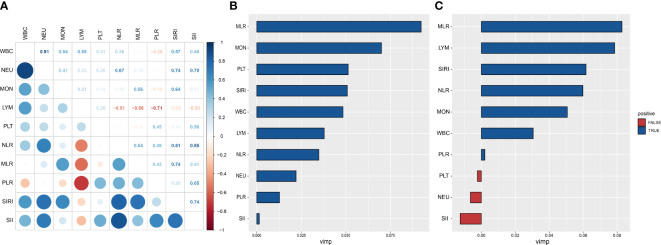
Prognostic value of complete blood cell count (CBC)-derived indicators. Spearman correlation analysis was used to calculate the correlation coefficients among CBC parameters and CBC-derived inflammatory biomarkers **(A)**. A random subsistence forest method was used to compare the value of CBC parameters and CBC-derived inflammatory biomarkers in predicting all-cause **(B)** and respiratory disease **(C)** mortality in adults with asthma.

We examined the correlation between CBC parameters and CBC-derived inflammatory biomarkers (**Figure 3A**). There was a significant positive correlation between platelet and SII (r=0.85) and a significant negative correlation between lymphocyte and PLR (r=-0.71). We utilized a RSF analysis to compare the prognostic value of complete blood count (CBC) parameters and CBC-derived inflammatory biomarkers for predicting all-cause and respiratory disease mortality in adults with asthma. Our findings demonstrated that among the CBC-derived inflammatory biomarkers, monocyte-to-lymphocyte ratio (MLR) had the highest prognostic value for both all-cause and respiratory disease mortality (**Figures 3B, C**). These results indicate that MLR is a strong predictor of mortality in this population and may have important clinical implications for risk stratification and disease management.

### 3.5 Stratified analysis

**Table S4** presents the results of the comprehensive stratified analyses examining the associations between quartiles of CBC-derived inflammatory biomarkers levels and all-cause and respiratory disease mortality by smoking among adults with asthma. Stratified analysis revealed a significant association between higher levels of CBC-derived inflammatory biomarkers and increased risk of all-cause and respiratory disease mortality in both subgroups, the smoking and never-smoking groups. Thus, smoking status did not have a significant effect on the relationship between whole blood cell-derived inflammatory biomarkers and the risk of mortality (all *P* for interaction > 0.05).

### 3.6 Sensitivity analyses

In sensitivity analyses, we further adjusted the full model for anti-asthma medications as well as lung function parameters, and the results similarly showed that CBC-derived inflammatory markers were associated with all-cause mortality and respiratory disease mortality (**Tables S5**, **S6**). If participants with adult asthma who died during the first two years of follow-up were excluded, we observed similar results in the adjusted full model (**Table S7**). In addition, after participants with a history of cancer, HIV infection, or respiratory infection at baseline were also excluded from the study, a repeat COX regression similarly showed that higher CBC-derived inflammatory markers were associated with an increased risk of all-cause and respiratory disease mortality (**Table S8**). Finally, if we exclude participants with self-reported COPD at baseline from the study, the relationship remains (**Table S9**).

## 4 Discussion

We conducted a cross-sectional study of 48,305 adults to investigate the association between CBC-derived inflammatory biomarkers and the prevalence and mortality risk of asthma in the US population from 1999 to 2018. Our findings indicate that NLR, PLR, MLR, SIRI, and SII were positively associated with the prevalence of asthma in the population studied. We conducted a longitudinal cohort study of 6,403 individuals with asthma to investigate the association between CBC-derived inflammatory biomarkers and mortality risk. After adjusting for relevant confounding factors, we found that higher levels of NLR, MLR, SIRI, and SII were significantly associated with increased risk of all-cause and respiratory disease mortality in adults with asthma. Furthermore, our results from the random survival forest analysis demonstrated that MLR was the most predictive biomarker of mortality risk in this population. These findings suggest that CBC-derived inflammatory biomarkers, particularly MLR, may be useful clinical tools for assessing mortality risk in individuals with asthma. Overall, our study highlights the importance of considering the inflammatory status reflected by CBC-derived markers as an independent risk factor for all-cause and respiratory disease mortality in individuals with asthma.

Asthma is a chronic inflammatory disease of the airways involving multiple cells (eosinophils, neutrophils, lymphocytes, and macrophages) and cellular components (26, 27). In the past, many studies have focused on the role of eosinophils as blood biomarkers for asthma (28). Recently, CBC parameters are recommended as supplemental markers for asthma core outcome (29). In our study, the counts of WBC, neutrophils, monocyte were significantly associated with a higher prevalence of asthma. In addition, we found that neutrophils and monocyte were significantly associated with a higher risk of all-cause and respiratory disease mortality. Girdhar et al. (30) found that the total number of WBC in plasma was negatively correlated with lung function in asthma patients, which supported our view. Patients with asthma persistence were older and had higher levels of blood neutrophils as compared to patients who experienced clinical remission (31). Aberrant differentiation of monocytes and increased monocyte-derived TGF-β1 can identify severe asthma patients (32).

CBC-derived indicators are markers that reflect the immune and chronic inflammatory states of the body (33, 34), which have been studied in recent years (35, 36). The role of CBC-derived indicators in respiratory diseases has aroused strong interest among researchers. Citu et al. (37) identified elevated NLR and MLR as independent factors for poor clinical outcome of COVID-19. In agreement with previous studies (15, 38), Fois et al. (39) found increased values of NLR in severe COVID-19 disease patients. However, they also reported that other CBC-derived inflammation indexes, such as SII and SIRI are also significantly associated with disease severity. The patients with PLR, NLR, MLR, and SII above the cut-off had a survival probability of COVID-19 50% lower (14).

However, the role of CBC-derived indicators in the diagnosis and prognosis of asthma is not clear. A meta-analysis showed that the NLR values are a reasonable and easy-to-use marker for asthma and its exacerbations (40). Shi et al. showed that NLR, as a non-specific inflammatory index, can effectively diagnose patients with an acute attack of asthma and is related to the severity of the disease (41). Compared to lower SII levels, higher SII levels increased mortality risk in people with asthma (19). In our study, NLR, MLR, SIRI and SII were significantly associated with the prevalence of asthma and increased the risk of all-cause and respiratory disease mortality in patients with asthma. In addition, MLR was proved to be an independent predictor of prognosis in patients with asthma. MLR is calculated based on monocyte and lymphocyte counts, so an elevated MLR indicates an increase in monocytes. Studies have shown that monocytes promote inflammatory processes by differentiating into macrophages or dendritic cells, which can impair lung function and promote asthma (42–45). We speculate that increased MLR levels predict a poor outcome for asthma.

Our study revealed that among the five blood-derived inflammatory markers analyzed, the MLR emerged as the most valuable predictor of mortality in participants with asthma. Asthma, a chronic inflammatory disorder of the airways, is associated with complex immunological processes (46). MLR reflects the interplay between innate and adaptive immune responses (47–49). Elevated MLR levels have been shown to be indicative of increased systemic inflammation and immune dysregulation (50). In the context of asthma, MLR holds particular significance due to its potential role in capturing the dynamic balance between pro-inflammatory monocytes and anti-inflammatory lymphocytes. The higher MLR values observed in individuals who experience adverse outcomes suggest a skewed immune response towards a more pro-inflammatory state (51–53). This dysregulation in the MLR may contribute to the progression of inflammation, exacerbation of airway hyperresponsiveness, and ultimately, increased mortality risk in asthma patients (42–45). These findings highlight the relevance of MLR as a comprehensive inflammatory marker, encompassing both innate and adaptive immunity, and its potential to provide valuable insights into the underlying biological mechanisms contributing to asthma mortality.

Our study has several advantages compared to previous investigations. Firstly, our large sample size enabled us to detect significant associations between CBC-derived indicators and both the prevalence of asthma and mortality risk in participants. Secondly, we adjusted for several key confounding factors that may have affected our results, including personal habits, personal history, and socioeconomic status. Thirdly, CBC-derived indicators are composed of multiple CBC parameters, which may provide more comprehensive information than single indices, making them valuable tools in clinical settings for the management and treatment of asthma in adults. Lastly, we employed a robust analysis method, RSF, which overcomes the issue of high collinearity between inflammatory biomarkers and identified MLR as the most valuable predictor of all-cause and respiratory disease mortality in adults with asthma. Overall, our study provides novel insights into the prognostic value of CBC-derived indicators in asthma and has important implications for clinical management and future research in this field.

This study also has several limitations. First, the NHANES findings were based on the self-reports of the patients, which may lead to recall bias. Second, the results of the studies were adjusted for age, sex, smoking status, and other confounding factors, but there may be unknown confounding factors affecting the analyses. Third, we calculated CBC-derived indicators with one-time CBC parameters which might cause bias.

## 5 Conclusions

The findings of this study suggest that among adults with asthma, increased levels of NLR, PLR, SIRI, and SII are associated with higher risks of all-cause and respiratory disease mortality. The measurement of MLR may serve as a useful tool for identifying high-risk adults with asthma in a simple and cost-effective manner. These findings contribute to the growing body of evidence on the potential utility of these biomarkers in predicting asthma outcomes and may inform clinical decision-making in the management of asthma patients.

## Data availability statement

Publicly available datasets were analyzed in this study. This data can be found here: https://www.cdc.gov/nchs/nhanes.

## Ethics statement

The studies involving human participants were reviewed and approved by The National Center for Health Statistics (NCHS) Research Ethics Review Board. The patients/participants provided their written informed consent to participate in this study.

## Author contributions

The authors’ responsibilities were as follows—SW: designed the research, and had primary responsibility for the final content; JK: conducted analyses and wrote the first draft of the paper; FQ and WF: revised the manuscript; All authors contributed to the article and approved the submitted version.
