# Supplementary material for: Associations of complete blood cell count-derived inflammatory biomarkers with asthma and mortality in adults: a population-based study
Source: Front Immunol. 2023 Jul 28;14:1205687. doi: 10.3389/fimmu.2023.1205687 (PMC10416440; doi:10.3389/fimmu.2023.1205687)
Supplement: Supplementary file 1 [file DataSheet_1.docx]

**
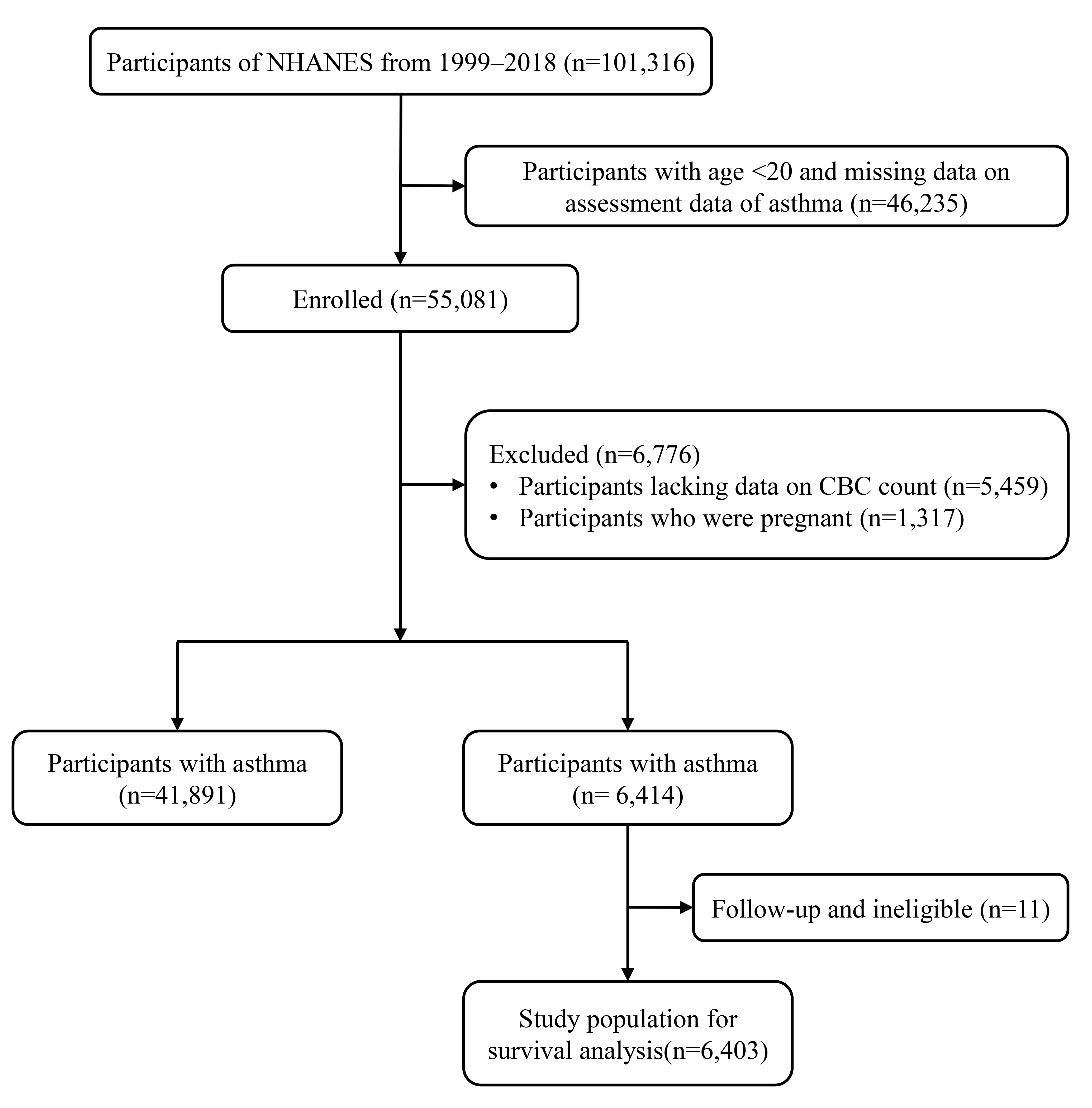
Online Supplementary Material**

**Figure S1.** Flowchart of the study participants

**Table S1.** OR (95% CIs) of the prevalence of asthma according to quartiles of complete blood cell (CBC) counts among adults in NHANES 1999–2018.

|  | Quartiles of CBC-derived inflammatory biomarkers levels | | | | *P*_trend_ |
| --- | --- | --- | --- | --- | --- |
|  | Quartile 1 | Quartile 2 | Quartile 3 | Quartile 4 |  |
| **WBC** | |  |  |  |  |
| Range | <5.7 | 5.7-6.9 | 7.0-8.4 | >8.4 |  |
| Crude | 1.00 [Reference] | 1.039(0.936,1.152) | 1.108(0.997,1.232) | 1.338(1.220,1.468) | <0.001 |
| Model 1 | 1.00 [Reference] | 1.060(0.956,1.176) | 1.123(1.008,1.250) | 1.335(1.214,1.468) | <0.001 |
| Model 2 | 1.00 [Reference] | 0.995(0.896,1.106) | 1.010(0.904,1.130) | 1.125(1.016,1.246) | 0.017 |
| **NEU** | |  |  |  |  |
| Range | <3.1 | 3.1-4.0 | 4.1-5.1 | >5.1 |  |
| Crude | 1.00 [Reference] | 1.066(0.959,1.186) | 1.085(0.980,1.201) | 1.289(1.166,1.425) | <0.001 |
| Model 1 | 1.00 [Reference] | 1.101(0.988,1.227) | 1.117(1.008,1.238) | 1.305(1.177,1.448) | <0.001 |
| Model 2 | 1.00 [Reference] | 1.044(0.935,1.165) | 1.016(0.913,1.131) | 1.119(1.001,1.250) | 0.047 |
| **MON** |  |  |  |  |  |
| Range | <0.4 | 0.4-0.5 | 0.6-0.7 | >0.7 |  |
| Crude | 1.00 [Reference] | 1.002(0.913,1.099) | 1.040(0.952,1.136) | 1.163(1.033,1.308) | 0.01 |
| Model 1 | 1.00 [Reference] | 1.033(0.941,1.134) | 1.117(1.022,1.221) | 1.281(1.134,1.446) | <0.001 |
| Model 2 | 1.00 [Reference] | 1.002(0.912,1.101) | 1.054(0.964,1.153) | 1.153(1.017,1.307) | 0.018 |
| **LYM** |  |  |  |  |  |
| Range | <1.6 | 1.6-2.0 | 2.1-2.5 | >2.5 |  |
| Crude | 1.00 [Reference] | 1.069(0.964,1.187) | 1.052(0.953,1.162) | 1.159(1.062,1.265) | 0.004 |
| Model 1 | 1.00 [Reference] | 1.026(0.922,1.142) | 0.990(0.892,1.098) | 1.068(0.975,1.170) | 0.249 |
| Model 2 | 1.00 [Reference] | 0.986(0.885,1.098) | 0.917(0.826,1.018) | 0.939(0.855,1.031) | 0.115 |
| **PLT** |  |  |  |  |  |
| Range | <207.0 | 207.0-245.0 | 246.0-289.0 | >289.0 |  |
| Crude | 1.00 [Reference] | 1.060(0.962,1.167) | 1.158(1.051,1.277) | 1.266(1.153,1.389) | <0.001 |
| Model 1 | 1.00 [Reference] | 0.994(0.902,1.094) | 1.055(0.953,1.167) | 1.096(0.994,1.208) | 0.036 |
| Model 2 | 1.00 [Reference] | 0.994(0.903,1.095) | 1.040(0.940,1.150) | 1.046(0.943,1.159) | 0.3 |

Data are presented as OR (95% CI) unless indicated otherwise; Model 1 was adjusted as age (continuous), sex (male or female), and race/ethnicity (Mexican American, Other Hispanic, Non-Hispanic White, Non-Hispanic Black or Other); Model 2 was adjusted as model 1 plus education level (below high school, high school, or above high school), family poverty income ratio (≤1.0, 1.1–3.0, or >3.0), drinking status (nondrinker, low-to-moderate drinker, or heavy drinker), smoking status (never smoker, former smoker, or current smoker), BMI (<25.0, 25.0-29.9, or >29.9), physical activity (inactive, insufficiently active, or active), total energy intakes (in quartiles), self-reported diabetes (yes or no), and self-reported hypertension (yes or no).

**Table S2.** Baseline characteristics of adults with asthma in NHANES 1999–2018.

| Characteristics | Total (n=6403) | | All-cause Mortality | | *P* value |
| --- | --- | --- | --- | --- | --- |
|  |  |  | No (n=5474) | Yes (n=929) |  |
| Age, years | 45.37(0.29) | | 43.19(0.29) | 63.86(0.56) | <0.001 |
| Male, % | 2708(42.29) | | 2269(41.31) | 439(41.82) | 0.820 |
| Race/ethnicity, % |  | |  |  | 0.010 |
| Mexican American | 656(10.25) | | 581(5.07) | 75(2.42) |  |
| Other Hispanic | 590(9.21) | | 535(6.04) | 55(5.39) |  |
| Non-Hispanic White | 3083(48.15) | | 2529(69.79) | 554(76.23) |  |
| Non-Hispanic Black | 1523(23.79) | | 1318(12.23) | 205(11.77) |  |
| Other race | 551(8.61) | | 511(6.87) | 40(4.20) |  |
| Education level, % |  | |  |  | <0.001 |
| Below high school | 1503(23.47) | | 1148(14.12) | 355(29.94) |  |
| High school | 1439(22.47) | | 1219(22.41) | 220(25.91) |  |
| Above high school | 3461(54.05) | | 3107(63.47) | 354(44.15) |  |
| Family PIR, % |  | |  |  | <0.001 |
| ≤1.0 | 1602(25.02) | | 1335(17.30) | 267(25.87) |  |
| 1.1–3.0 | 2602(40.64) | | 2147(34.72) | 455(45.87) |  |
| >3.0 | 2199(34.34) | | 1992(47.98) | 207(28.27) |  |
| Smoking status, % |  | |  |  | <0.001 |
| Never smoker | 3129(48.87) | | 2830(51.54) | 299(31.31) |  |
| Former smoker | 1696(26.49) | | 1322(24.70) | 374(37.89) |  |
| Current smoker | 1578(24.64) | | 1322(23.75) | 256(30.80) |  |
| Drinking status, % |  | |  |  | <0.001 |
| Nondrinker | 1381(21.57) | | 1120(16.47) | 261(27.94) |  |
| Low-to-moderate drinker | 4518(70.56) | | 3922(74.82) | 596(62.58) |  |
| Heavy drinker | 504(7.87) | | 432(8.71) | 72(9.48) |  |
| Body mass index, % |  | |  |  | 0.190 |
| <25.0 kg/m^2^ | 1626(25.39) | | 1393(28.69) | 233(24.98) |  |
| 25.0-29.9 kg/m^2^ | 1850(28.89) | | 1561(28.82) | 289(29.51) |  |
| >29.9 kg/m^2^ | 2927(45.71) | | 2520(42.48) | 407(45.51) |  |
| Physical activity, % |  | |  |  | <0.001 |
| Inactive | 1821(28.44) | | 1357(21.14) | 464(47.85) |  |
| Insufficiently active | 2259(35.28) | | 2008(39.42) | 251(27.50) |  |
| Active | 2323(36.28) | | 2109(39.45) | 214(24.65) |  |
| Total energy intakes, kcal/day | 1994.00(1467.00,2736.00) | | 2031.00(1493.00,2781.00) | 1620.00(1244.78,2210.00) | <0.001 |
| Self-reported hypertension, % | 2648(41.36) | | 2034(31.80) | 614(62.59) | <0.001 |
| Self-reported diabetes, % | 975(15.23) | | 716(8.92) | 259(25.57) | <0.001 |
| Anti-asthmatic drugs, % |  | |  |  |  |
| Bronchodilator | 1545(24.14) | | 1178(20.70) | 367(40.73) | <0.001 |
| Inhaled corticosteroids | 817(12.77) | | 614(11.00) | 203(22.41) | <0.001 |
| Others | 645(10.08) | | 463(9.07) | 182(21.12) | <0.001 |
| CBC count, 10^3^/μL |  | |  |  |  |
| White blood cell | 7.20(5.90,8.70) | | 7.10(5.90,8.60) | 7.50(6.10,9.10) | <0.001 |
| Neutrophils | 4.20(3.20,5.40) | | 4.10(3.20,5.30) | 4.60(3.50,5.80) | <0.001 |
| Monocyte | 0.50(0.40,0.70) | | 0.50(0.40,0.70) | 0.60(0.50,0.70) | <0.001 |
| Lymphocyte | 2.10(1.70,2.60) | | 2.10(1.70,2.60) | 1.90(1.40,2.40) | <0.001 |
| Platelet | 251.00(213.00,297.00) | | 251.00(214.00,296.00) | 253.00(203.00,303.00) | 0.570 |
| CBC-derived indicators | |  | | |  |
| NLR | 2.00(1.54,2.65) | | 1.97(1.52,2.59) | 2.35(1.73,3.44) | <0.001 |
| PLR | 121.18(96.79,154.38) | | 119.57(96.43,152.50) | 132.78(101.90,174.38) | <0.001 |
| MLR | 0.26(0.21,0.33) | | 0.25(0.20,0.33) | 0.31(0.23,0.44) | <0.001 |
| SIRI, 10^3^/μL | 1.08(0.76,1.58) | | 1.06(0.74,1.51) | 1.37(0.95,2.25) | <0.001 |
| SII, 10^3^/μL | 506.40(366.33,705.55) | | 497.45(361.54,690.38) | 605.50(411.80,860.71) | <0.001 |

Abbreviations: PIR, poverty income ratio; NLR, neutrophil-to-lymphocyte ratio; PLR, platelet-to- lymphocyte ratio; MLR, monocyte-to-lymphocyte ratio; SIRI, systemic inflammatory response index; SII, systemic immune-inflammation index; CBC, complete blood cell.

Normally distributed continuous variables are described as means ± SEs, and continuous variables without a normal distribution are presented as medians [interquartile ranges]. Categorical variables are presented as numbers (percentages). N reflect the study sample while percentages reflect the survey-weighted.

**Table S3.** HRs (95% CIs) of all-cause and respiratory disease mortality according to quartiles of complete blood cell (CBC) counts among adults with asthma in NHANES 1999–2018.

|  | Quartiles of CBC-derived inflammatory biomarkers levels | | | |  |
| --- | --- | --- | --- | --- | --- |
|  | Quartile 1 | Quartile 2 | Quartile 3 | Quartile 4 | *P* _trend_ |
| **All-cause mortality** | |  |  |  |  |
| WBC | 1 [Reference] | 0.945(0.753,1.185) | 1.022(0.788,1.326) | 1.166(0.921,1.477) | 0.161 |
| NEU | 1 [Reference] | 1.128(0.897,1.420) | 1.177(0.908,1.525) | 1.364(1.066,1.745) | 0.016 |
| MON | 1 [Reference] | 1.193(0.935,1.523) | 1.279(1.000,1.635) | 1.604(1.208,2.129) | 0.001 |
| LYM | 1 [Reference] | 0.683(0.565,0.826) | 0.655(0.510,0.840) | 0.749(0.581,0.966) | 0.004 |
| PLT | 1 [Reference] | 0.749(0.585,0.960) | 0.879(0.690,1.120) | 0.878(0.699,1.102) | 0.486 |
| **Respiratory disease mortality** | |  |  |  |  |
| WBC | 1 [Reference] | 1.583(0.830,3.018) | 2.026(1.101,3.728) | 2.724(1.498,4.952) | <0.001 |
| NEU | 1 [Reference] | 1.566(0.809, 3.030) | 2.041(1.112, 3.745) | 3.378(1.851, 6.163) | <0.0001 |
| MON | 1 [Reference] | 1.304(0.653, 2.602) | 1.475(0.782, 2.780) | 1.894(1.028, 3.489) | 0.036 |
| LYM | 1 [Reference] | 1.033(0.474, 2.251) | 1.346(0.664, 2.729) | 1.994(1.036, 3.837) | 0.010 |
| PLT | 1 [Reference] | 1.357(0.706, 2.610) | 1.912(1.170, 3.126) | 2.447(1.298, 4.616) | 0.002 |

Data are presented as HR (95% CI) unless indicated otherwise;

Model was adjusted as age (continuous), sex (male or female), race/ethnicity (Mexican American, Other Hispanic, Non-Hispanic White, Non-Hispanic Black or Other), education level (below high school, high school, or above high school), family poverty income ratio (≤1.0, 1.1–3.0, or >3.0), drinking status (nondrinker, low-to-moderate drinker, or heavy drinker), smoking status (never smoker, former smoker, or current smoker), BMI (<25.0, 25.0-29.9, or >29.9), physical activity (inactive, insufficiently active, or active), total energy intakes (in quartiles), self-reported diabetes (yes or no), and self-reported hypertension (yes or no).

**Table S4.** Stratified analyses of the associations between quartiles of CBC-derived inflammatory biomarkers levels and all-cause and respiratory disease mortality by smoking among adults with asthma in NHANES 1999–2018 (n=6403).

|  | Quartiles of CBC-derived inflammatory biomarkers levels | | | |  |
| --- | --- | --- | --- | --- | --- |
|  | Quartile 1 | Quartile 2 | Quartile 3 | Quartile 4 | *P* _trend_ |
| **Never smoker (n=3129)** | |  |  |  |  |
| All-cause mortality |  |  |  |  |  |
| NLR | 1 [Reference] | 1.584(1.045,2.400) | 1.472(0.907,2.391) | 2.286(1.442,3.624) | <0.001 |
| MLR | 1 [Reference] | 0.926(0.527,1.628) | 0.947(0.563,1.595) | 1.735(1.085,2.773) | 0.001 |
| PLR | 1 [Reference] | 1.221(0.737,2.023) | 1.276(0.834,1.953) | 1.382(0.837,2.282) | 0.203 |
| SIRI | 1 [Reference] | 1.074(0.618,1.869) | 1.280(0.767,2.136) | 2.106(1.284,3.456) | <0.001 |
| SII | 1 [Reference] | 1.175(0.750,1.841) | 1.070(0.715,1.601) | 1.561(1.054,2.310) | 0.028 |
| Respiratory disease mortality | |  |  |  |  |
| NLR | 1 [Reference] | 1.243(0.288, 5.359) | 1.556(0.269, 9.019) | 11.583(2.895, 46.338) | <0.001 |
| MLR | 1 [Reference] | 0.729(0.081, 6.586) | 1.037(0.103, 10.443) | 3.675(0.366, 36.946) | 0.045 |
| PLR | 1 [Reference] | 0.171(0.021, 1.362) | 0.910(0.191, 4.337) | 3.183(0.900, 11.262) | 0.015 |
| SIRI | 1 [Reference] | 4.048(0.550, 29.818) | 4.010(0.419, 38.354) | 18.058(2.294,142.177) | <0.001 |
| SII | 1 [Reference] | 1.955(0.408, 9.361) | 2.658(0.568, 12.449) | 7.299(1.934, 27.547) | 0.002 |
| **Former and current smoker (n=3274)** | | |  |  |  |
| All-cause mortality | |  |  |  |  |
| NLR | 1 [Reference] | 0.933(0.675,1.291) | 0.919(0.674,1.253) | 1.557(1.208,2.008) | <0.001 |
| MLR | 1 [Reference] | 1.230(0.864,1.753) | 1.211(0.834,1.759) | 1.678(1.212,2.323) | <0.001 |
| PLR | 1 [Reference] | 0.723(0.540,0.968) | 0.711(0.519,0.975) | 1.042(0.792,1.370) | 0.228 |
| SIRI | 1 [Reference] | 1.149(0.787,1.677) | 1.345(0.948,1.908) | 1.846(1.341,2.542) | <0.001 |
| SII | 1 [Reference] | 1.105(0.824,1.480) | 1.119(0.817,1.532) | 1.395(1.067,1.826) | 0.008 |
| Respiratory disease mortality | |  |  |  |  |
| NLR | 1 [Reference] | 0.681(0.276, 1.678) | 1.018(0.487, 2.129) | 2.209(1.120, 4.360) | <0.001 |
| MLR | 1 [Reference] | 1.255(0.492, 3.199) | 1.713(0.748, 3.921) | 2.713(1.413, 5.212) | <0.001 |
| PLR | 1 [Reference] | 0.424(0.197, 0.911) | 0.683(0.329, 1.416) | 1.030(0.552, 1.925) | 0.315 |
| SIRI | 1 [Reference] | 0.594(0.229, 1.541) | 1.735(0.765, 3.932) | 3.062(1.509, 6.213) | <0.001 |
| SII | 1 [Reference] | 0.674(0.279, 1.627) | 0.969(0.431, 2.180) | 1.823(0.942, 3.528) | 0.005 |

Data are presented as HR (95% CI) unless indicated otherwise;

Model was adjusted as age (continuous), sex (male or female), race/ethnicity (Mexican American, Other Hispanic, Non-Hispanic White, Non-Hispanic Black or Other), education level (below high school, high school, or above high school), family poverty income ratio (≤1.0, 1.1–3.0, or >3.0), drinking status (nondrinker, low-to-moderate drinker, or heavy drinker), smoking status (never smoker, former smoker, or current smoker), BMI (<25.0, 25.0-29.9, or >29.9), physical activity (inactive, insufficiently active, or active), total energy intakes (in quartiles), self-reported diabetes (yes or no), and self-reported hypertension (yes or no).

**Table S5.** HRs (95% CIs) of all-cause and respiratory disease mortality according to quartiles of CBC-derived inflammatory biomarkers levels among adults with asthma with further adjustment of anti-asthmatic drugs in NHANES 1999–2018 (n=6403).

|  | Quartiles of CBC-derived inflammatory biomarkers levels | | | |  |
| --- | --- | --- | --- | --- | --- |
|  | Quartile 1 | Quartile 2 | Quartile 3 | Quartile 4 | *P* _trend_ |
| **All-cause mortality** |  |  |  |  |  |
| NLR | 1 [Reference] | 1.144(0.899,1.458) | 1.075(0.840,1.376) | 1.733(1.347,2.228) | <0.001 |
| MLR | 1 [Reference] | 1.094(0.788,1.520) | 1.145(0.840,1.561) | 1.699(1.301,2.220) | <0.001 |
| PLR | 1 [Reference] | 0.853(0.676,1.076) | 0.954(0.757,1.201) | 1.151(0.919,1.441) | 0.073 |
| SIRI | 1 [Reference] | 1.092(0.805,1.480) | 1.301(0.970,1.745) | 1.758(1.319,2.343) | <0.001 |
| SII | 1 [Reference] | 1.145(0.890,1.472) | 1.148(0.895,1.472) | 1.389(1.100,1.754) | 0.006 |
| **Respiratory disease mortality** | |  |  |  |  |
| NLR | 1 [Reference] | 0.759(0.341,1.689) | 1.076(0.532, 2.175) | 2.401(1.255, 4.594) | <0.001 |
| MLR | 1 [Reference] | 1.055(0.452, 2.464) | 1.693(0.814, 3.521) | 2.654(1.425, 4.944) | <0.001 |
| PLR | 1 [Reference] | 0.426(0.210, 0.864) | 0.893(0.452, 1.765) | 1.283(0.759, 2.169) | 0.064 |
| SIRI | 1 [Reference] | 0.798(0.336, 1.893) | 1.765(0.800, 3.895) | 2.945(1.412, 6.141) | <0.001 |
| SII | 1 [Reference] | 0.671(0.284, 1.585) | 1.125(0.551, 2.299) | 1.719(0.905, 3.265) | 0.006 |

Data are presented as HR (95% CI) unless indicated otherwise;

Model was adjusted as age (continuous), sex (male or female), race/ethnicity (Mexican American, Other Hispanic, Non-Hispanic White, Non-Hispanic Black or Other), education level (below high school, high school, or above high school), family poverty income ratio (≤1.0, 1.1–3.0, or >3.0), drinking status (nondrinker, low-to-moderate drinker, or heavy drinker), smoking status (never smoker, former smoker, or current smoker), BMI (<25.0, 25.0-29.9, or >29.9), physical activity (inactive, insufficiently active, or active), total energy intakes (in quartiles), self-reported diabetes (yes or no), self-reported hypertension (yes or no) and anti-asthmatic drugs (bronchodilator, inhaled corticosteroids or other anti-asthmatic drugs).

**Table S6.** HRs (95% CIs) of all-cause mortality according to quartiles of CBC-derived inflammatory biomarkers levels among adults with asthma with further adjustment of lung function in NHANES 2009–2012 (n=1203).

|  | Quartiles of CBC-derived inflammatory biomarkers levels | | | |  |
| --- | --- | --- | --- | --- | --- |
|  | Quartile 1 | Quartile 2 | Quartile 3 | Quartile 4 | *P* _trend_ |
| NLR | 1 [Reference] | 1.413(0.697,2.861) | 0.993(0.348,2.833) | 2.786(1.272,6.100) | 0.004 |
| MLR | 1 [Reference] | 2.634(1.247,5.564) | 1.951(0.702,5.422) | 2.852(1.304,6.237) | 0.030 |
| PLR | 1 [Reference] | 0.926(0.386,2.221) | 1.701(0.881,3.284) | 1.666(0.857,3.240) | 0.080 |
| SIRI | 1 [Reference] | 2.065(0.988,4.318) | 1.977(0.875,4.467) | 2.237(1.279,3.910) | 0.014 |
| SII | 1 [Reference] | 1.218(0.566,2.622) | 1.111(0.504,2.446) | 1.677(0.900,3.127) | 0.069 |

Data are presented as HR (95% CI) unless indicated otherwise;

Model was adjusted as age (continuous), sex (male or female), race/ethnicity (Mexican American, Other Hispanic, Non-Hispanic White, Non-Hispanic Black or Other), education level (below high school, high school, or above high school), family poverty income ratio (≤1.0, 1.1–3.0, or >3.0), drinking status (nondrinker, low-to-moderate drinker, or heavy drinker), smoking status (never smoker, former smoker, or current smoker), BMI (<25.0, 25.0-29.9, or >29.9), physical activity (inactive, insufficiently active, or active), total energy intakes (in quartiles), self-reported diabetes (yes or no), self-reported hypertension (yes or no), forced vital capacity (continuous), and forced expiratory volume 1st second (continuous).

**Table S7.** HRs (95% CIs) of all-cause and respiratory disease mortality according to quartiles of CBC-derived inflammatory biomarkers levels among adults with asthma after excluding participants who died within two years of follow-up in NHANES 1999–2018 (n=6262).

|  | Quartiles of CBC-derived inflammatory biomarkers levels | | | |  |
| --- | --- | --- | --- | --- | --- |
|  | Quartile 1 | Quartile 2 | Quartile 3 | Quartile 4 | *P* _trend_ |
| **All-cause mortality** | |  |  |  |  |
| NLR | 1 [Reference] | 1.123(0.868,1.452) | 1.036(0.786,1.366) | 1.583(1.214,2.063) | <0.001 |
| MLR | 1 [Reference] | 1.076(0.758,1.526) | 1.160(0.835,1.610) | 1.592(1.214,2.086) | <0.0001 |
| PLR | 1 [Reference] | 0.841(0.645,1.097) | 0.912(0.703,1.184) | 1.096(0.844,1.424) | 0.167 |
| SIRI | 1 [Reference] | 1.141(0.822,1.583) | 1.285(0.941,1.754) | 1.610(1.194,2.171) | <0.001 |
| SII | 1 [Reference] | 1.092(0.838,1.424) | 1.096(0.828,1.452) | 1.316(1.023,1.695) | 0.021 |
| **Respiratory disease mortality** | |  |  |  |  |
| NLR | 1 [Reference] | 0.567(0.239, 1.345) | 0.937(0.463, 1.897) | 2.292(1.191, 4.410) | <0.0001 |
| MLR | 1 [Reference] | 1.181(0.492, 2.836) | 1.558(0.708, 3.426) | 2.812(1.450, 5.452) | <0.001 |
| PLR | 1 [Reference] | 0.461(0.226, 0.942) | 0.792(0.378, 1.661) | 1.219(0.707, 2.103) | 0.095 |
| SIRI | 1 [Reference] | 0.757(0.323, 1.777) | 1.637(0.748, 3.581) | 2.746(1.327, 5.681) | <0.0001 |
| SII | 1 [Reference] | 0.867(0.381, 1.976) | 0.966(0.424, 2.203) | 1.978(0.997, 3.922) | 0.006 |

Data are presented as HR (95% CI) unless indicated otherwise;

Model was adjusted as age (continuous), sex (male or female), race/ethnicity (Mexican American, Other Hispanic, Non-Hispanic White, Non-Hispanic Black or Other), education level (below high school, high school, or above high school), family poverty income ratio (≤1.0, 1.1–3.0, or >3.0), drinking status (nondrinker, low-to-moderate drinker, or heavy drinker), smoking status (never smoker, former smoker, or current smoker), BMI (<25.0, 25.0-29.9, or >29.9), physical activity (inactive, insufficiently active, or active), total energy intakes (in quartiles), self-reported diabetes (yes or no), and self-reported hypertension (yes or no).

**Table S8.** HRs (95% CIs) of all-cause and respiratory disease mortality according to quartiles of CBC-derived inflammatory biomarkers levels among adults with asthma after excluding participants with a history of cancer, HIV infection, or respiratory infection at baseline in NHANES 1999–2018 (n=5341).

|  | Quartiles of CBC-derived inflammatory biomarkers levels | | | |  |
| --- | --- | --- | --- | --- | --- |
|  | Quartile 1 | Quartile 2 | Quartile 3 | Quartile 4 | *P* _trend_ |
| **All-cause mortality** | |  |  |  |  |
| NLR | 1 [Reference] | 1.207(0.907,1.607) | 0.972(0.723,1.307) | 1.616(1.220,2.142) | <0.001 |
| MLR | 1 [Reference] | 1.158(0.829,1.616) | 0.996(0.698,1.423) | 1.608(1.178,2.194) | <0.001 |
| PLR | 1 [Reference] | 0.836(0.629,1.111) | 0.840(0.623,1.132) | 1.068(0.810,1.407) | 0.303 |
| SIRI | 1 [Reference] | 0.831(0.614,1.126) | 1.161(0.816,1.650) | 1.467(1.074,2.002) | <0.001 |
| SII | 1 [Reference] | 1.108(0.842,1.457) | 1.142(0.846,1.540) | 1.353(1.029,1.777) | 0.028 |
| **Respiratory disease mortality** | |  |  |  |  |
| NLR | 1 [Reference] | 0.811(0.306, 2.147) | 1.069(0.455, 2.510) | 2.728(1.259, 5.909) | <0.001 |
| MLR | 1 [Reference] | 1.349(0.503, 3.619) | 1.008(0.402, 2.523) | 3.088(1.326, 7.191) | <0.001 |
| PLR | 1 [Reference] | 0.624(0.280, 1.390) | 0.913(0.400, 2.087) | 1.462(0.737, 2.904) | 0.113 |
| SIRI | 1 [Reference] | 0.697(0.271, 1.794) | 1.578(0.590, 4.225) | 2.697(1.129, 6.444) | <0.001 |
| SII | 1 [Reference] | 1.070(0.435, 2.631) | 0.885(0.386, 2.026) | 2.164(0.980, 4.780) | 0.037 |

Data are presented as HR (95% CI) unless indicated otherwise;

Model was adjusted as age (continuous), sex (male or female), race/ethnicity (Mexican American, Other Hispanic, Non-Hispanic White, Non-Hispanic Black or Other), education level (below high school, high school, or above high school), family poverty income ratio (≤1.0, 1.1–3.0, or >3.0), drinking status (nondrinker, low-to-moderate drinker, or heavy drinker), smoking status (never smoker, former smoker, or current smoker), BMI (<25.0, 25.0-29.9, or >29.9), physical activity (inactive, insufficiently active, or active), total energy intakes (in quartiles), self-reported diabetes (yes or no), and self-reported hypertension (yes or no).

**Table S9.** HRs (95% CIs) of all-cause and respiratory disease mortality according to quartiles of CBC-derived inflammatory biomarkers levels among adults with asthma after excluding participants who had COPD history at baseline in NHANES 2013–2018 (n=2019).

|  | Quartiles of CBC-derived inflammatory biomarkers levels | | | |  |
| --- | --- | --- | --- | --- | --- |
|  | Quartile 1 | Quartile 2 | Quartile 3 | Quartile 4 | *P* _trend_ |
| NLR | 1 [Reference] | 0.468(0.188,1.167) | 1.003(0.546,1.844) | 2.069(0.912,4.694) | 0.006 |
| MLR | 1 [Reference] | 0.741(0.234,2.347) | 1.824(0.567,5.867) | 2.342(0.973,5.635) | 0.016 |
| PLR | 1 [Reference] | 0.510(0.243,1.069) | 0.703(0.397,1.245) | 0.791(0.359,1.746) | 0.742 |
| SIRI | 1 [Reference] | 0.793(0.316,1.991) | 1.275(0.487,3.342) | 2.252(0.947,5.357) | 0.004 |
| SII | 1 [Reference] | 0.988(0.495,1.975) | 1.053(0.464,2.392) | 1.327(0.637,2.762) | 0.352 |

Data are presented as HR (95% CI) unless indicated otherwise;

Model was adjusted as age (continuous), sex (male or female), race/ethnicity (Mexican American, Other Hispanic, Non-Hispanic White, Non-Hispanic Black or Other), education level (below high school, high school, or above high school), family poverty income ratio (≤1.0, 1.1–3.0, or >3.0), drinking status (nondrinker, low-to-moderate drinker, or heavy drinker), smoking status (never smoker, former smoker, or current smoker), BMI (<25.0, 25.0-29.9, or >29.9), physical activity (inactive, insufficiently active, or active), total energy intakes (in quartiles), self-reported diabetes (yes or no), and self-reported hypertension (yes or no).
